# Wastewater-based epidemiology applied at the building-level reveals distinct virome profiles based on the age of the contributing individuals

**DOI:** 10.1186/s40246-024-00580-1

**Published:** 2024-02-01

**Authors:** Cristina Mejías-Molina, Anna Pico-Tomàs, Sandra Martínez-Puchol, Marta Itarte, Helena Torrell, Núria Canela, Carles M. Borrego, Lluís Corominas, Marta Rusiñol, Sílvia Bofill-Mas

**Affiliations:** 1 Laboratory of Viruses Contaminants of Water and Food, Genetics, Microbiology and Statistics Department, Universitat de Barcelona, Barcelona, Catalonia Spain; 2 The Water Research Institute (IdRA), Universitat de Barcelona, Barcelona, Catalonia Spain; 3 Catalan Institute for Water Research (ICRA), Girona, Spain; 4 Centre for Omic Sciences (COS), Joint Unit Universitat Rovira I Virgili-EURECAT, Unique Scientific and Technical Infrastructures (ICTS), Eurecat, Centre Tecnològic de Catalunya, Reus, Catalonia Spain; 5 Group of Molecular Microbial Ecology, Institute of Aquatic Ecology, University of Girona, Girona, Catalonia Spain

**Keywords:** Human age-related virome, Building-level, Passive samplers, Wastewater-based epidemiology, Targeted enrichment sequencing

## Abstract

**Background:**

Human viruses released into the environment can be detected and characterized in wastewater. The study of wastewater virome offers a consolidated perspective on the circulation of viruses within a population. Because the occurrence and severity of viral infections can vary across a person’s lifetime, studying the virome in wastewater samples contributed by various demographic segments can provide valuable insights into the prevalence of viral infections within these segments. In our study, targeted enrichment sequencing was employed to characterize the human virome in wastewater at a building-level scale. This was accomplished through passive sampling of wastewater in schools, university settings, and nursing homes in two cities in Catalonia. Additionally, sewage from a large urban wastewater treatment plant was analysed to serve as a reference for examining the collective excreted human virome.

**Results:**

The virome obtained from influent wastewater treatment plant samples showcased the combined viral presence from individuals of varying ages, with astroviruses and human bocaviruses being the most prevalent, followed by human adenoviruses, polyomaviruses, and papillomaviruses. Significant variations in the viral profiles were observed among the different types of buildings studied. Mamastrovirus 1 was predominant in school samples, salivirus and human polyomaviruses JC and BK in the university settings while nursing homes showed a more balanced distribution of viral families presenting papillomavirus and picornaviruses and, interestingly, some viruses linked to immunosuppression.

**Conclusions:**

This study shows the utility of building-level wastewater-based epidemiology as an effective tool for monitoring the presence of viruses circulating within specific age groups. It provides valuable insights for public health monitoring and epidemiological studies.

**Supplementary Information:**

The online version contains supplementary material available at 10.1186/s40246-024-00580-1.

## Background

Human viruses can be released into the environment through faeces, urine, saliva or desquamation of skin cells, [1] and, as other excreted substances, they can be identified and detected in wastewater. Analysing the sewage virome offers a significant understanding into the viruses that are actively circulating at a given population and has also the potential to elucidate the introduction of emerging viruses and their modes of transmission [2]. From the initial polio surveillance efforts in the early 1930s to the present-day COVID-19 pandemic, wastewater-based epidemiology (WBE) has been widely employed to monitor vaccine-derived viruses and evaluate population immunity in areas where inactivated virus vaccines have been administered [3].

Numerous studies have documented the virome of urban wastewater, revealing the coexistence of viral families associated with persistent infections (*Anelloviridae, Parvoviridae, Adenoviridae, Papillomaviridae, Polyomaviridae, Herpesviridae*) alongside commonly viral families that contribute more directly to viral disease burdens (including *Astroviridae, Picornaviridae, Coronaviridae*) [4, 5].

It should be noted that the occurrence and severity of viral infections can vary across different stages of an individual's life. School-age children generally experience milder symptoms compared to other age groups, while the severity of viral infections tends to increase well before reaching old age [6].

To the best of our knowledge, various studies have explored the application of WBE at the building level with a focus on SARS-CoV-2 occurrence [7–14]. However, the building scale has been hardly considered for virome studies. Notably, McCall et al. [15] recently reported distinct viral profiles in wastewater collected from different types of buildings. Obtaining representative wastewater samples at a building-scale is challenging, but passive sampling has recently reemerged as an effective solution to address this issue [13, 16].

Next-generation sequencing (NGS) techniques are evolving as valuable complements to traditional molecular methods for analysing environmental samples. NGS provides insights into viral sequences present in these samples without the need for prior information. In recent years, advancements in sample multiplexing, streamlined library preparation protocols and bioinformatic tools have made NGS a more practical and accessible approach for viral detection [17]. Although viral detection by NGS in complex samples such as wastewater is still challenging, targeted enrichment sequencing (TES) strategies have shown to be a promising tool for viral detection and discovery in wastewater [2, 18]

The objective of this work was to characterize the virome present in wastewater at building-level, specifically in schools, university settings and nursing homes hosting residents of different age’s ranges, social habits and levels of vulnerability. The goal was to assess how different age groups contribute with different viral profiles to wastewater. To achieve this, passive samplers were utilized to collect wastewater samples from schools, university settings and nursing homes in two different cities. These samples were then subjected to virome characterization using a TES approach. Obtained results were compared with the virome profile of a large urban wastewater treatment plant (WWTP), which served as a reference.

## Results

### Abundance and diversity of viral reads in different types of wastewater samples

**Fig. 1:**
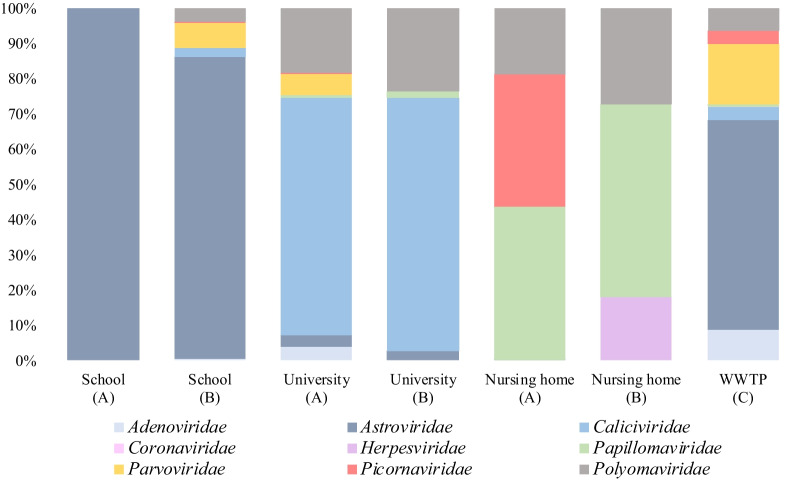
Relative abundance (% reads matching a viral family/total reads) of the detected viral families at building and WWTP-level representing different age-group wastewater contributors. **A**–**C** represents the different cities studied

**Fig. 2:**
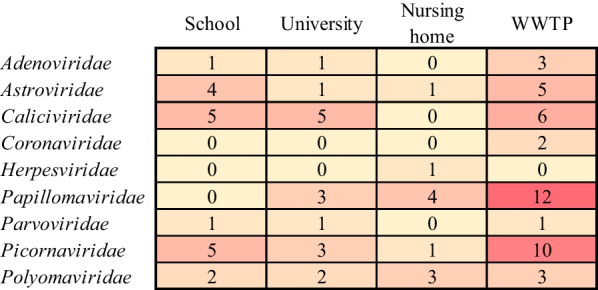
Heatmap profile showing the abundance of viral species detected in building and WWTP-level wastewater. Numbers indicate the quantity of different species that has, at least, one sequence with a positive BLAST hit that passed all the selection criteria. Data spanned from yellow (not detected) to red (high abundance)

Bioinformatic analysis of the raw data generated from TES applied to the four distinct wastewater types analysed yielded a total of 3,019,032 reads. These reads were taxonomically ascribed to 11 vertebrate viral families, 9 of which included families containing human viruses, encompassing 21 genera. Within the comprehensive analysis of wastewater samples, we identified a total of 48 distinct viral species, and in certain instances, we were able to further classify them into serotypes, serogroups, or genotypes. After manually trimming, a total of 142,643 human viral reads were obtained from the WWTP influent and the wastewater collected from schools, university settings, and nursery homes yielded 1,495,358, 50,520, and 142,643 human viral reads, respectively. Shannon indexes were calculated in the sites presenting a higher number of reads being of 1.77, 1.69, 1.48 and 1.31 in the WWTP, School (B), University (A) and Nursing Home (A), respectively. The relative abundances of different viral families comprising human viruses detected at each sampling site are depicted in Fig. 1, while the number of different viral species identified for each viral family is illustrated in Fig. 2.

### Wastewater treatment plant

**Fig. 3:**
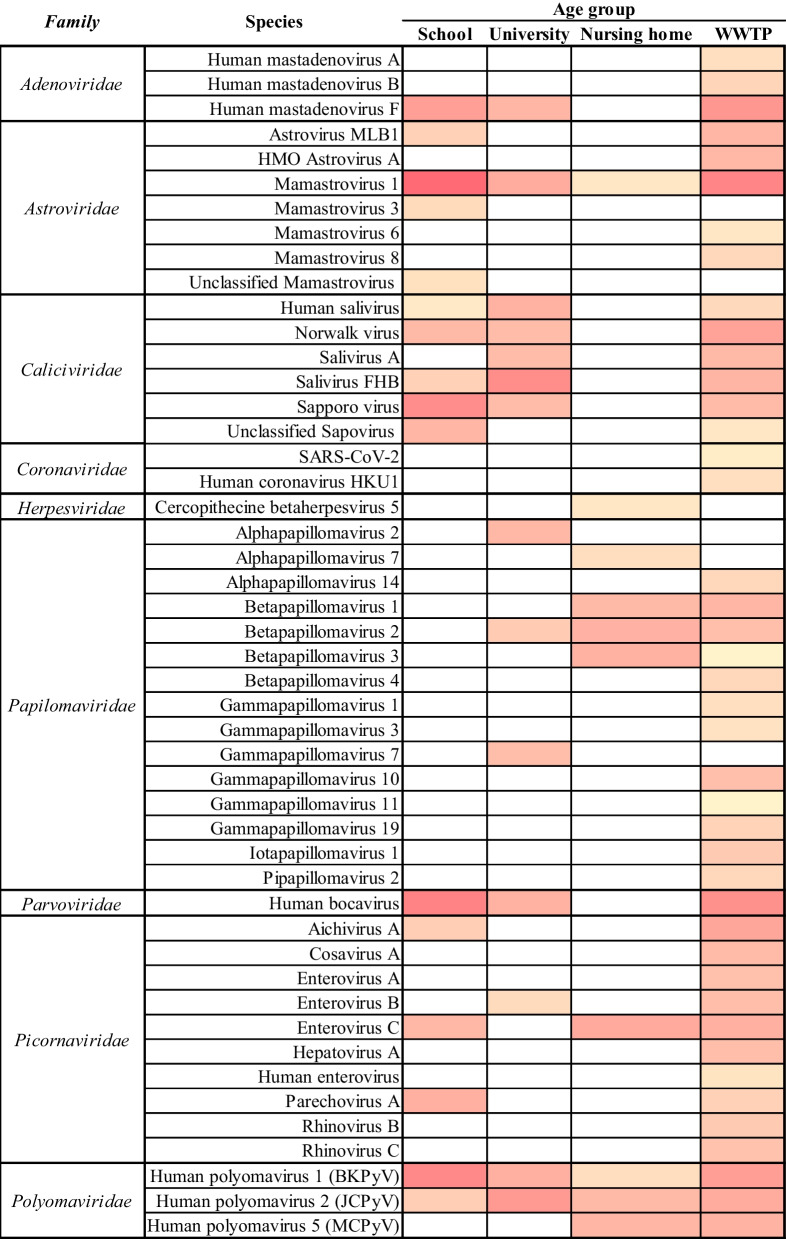
Relative viral species abundance (Log10 number of reads) detected at building and WWTP-level

Urban wastewater encompassed a wide range of viral families and viral species, displaying remarkable diversity (Fig. 3 and Additional file 1: Table S1). The higher number of reads were associated with the family *Astroviridae*, particularly with Mamastrovirus 1 (AstV-1). Notably, reads matching other *Astroviridae* species like AstV-6, AstV-8, and HMO-AstV-A were exclusively detected in urban wastewater, distinguishing it from the other wastewater samples analysed from the buildings.

The second-highest number of reads matched Human Bocavirus (*Parvoviridae)*. The third-highest number of reads corresponded to three different subgroups within the *Adenoviridae* family (AdV). Among these, AdV-40 and -41 (group F) exhibited the highest relative number of classified AdVs reads.

Less abundantly, we also detected sequences matching the persistently excreted viruses JC, BK and MC polyomaviruses (JCPyV, BKPyV and MCPyV) and 12 different species of human papillomaviruses, that together with identified members of the family *Picornaviridae* displayed the greatest diversity observed in these samples (Fig. 2). The majority of the *Picornaviridae* reads matched Aichivirus A and Enterovirus-C (EV-C) species. Among the observed *Caliciviridae* sequences, those corresponding to Norwalk Virus, Salivirus A (Sal-A), Salivirus FHB (Sal-FHB), Human Salivirus (HSalV) and Sapovirus (SapV) were the most prevalent. Finally, reads corresponding to the *Coronaviridae* family, including SARS-CoV-2 and Human Coronavirus HKU1, were also detected being WWTP the only site where this viral family was detected along this study.

### Schools

School wastewater primarily contained viruses associated with gastroenteritis. Among the viral reads detected in school samples, sequences matching members of the *Astroviridae* family were found to be predominant resulting in over 6 logs of reads. In the site A school, *Astroviridae* was the sole viral family with only a few reads identified. More in detail, a high number of reads matching AstV-1 were consistently found in schools, especially in samples collected in January. Sequences matching other AstV such as AstV-3 and AstV-MLB1 were also identified.

*Caliciviridae* and *Picornaviridae* were the two families presenting the highest number of viral species (Fig. 2). Members of the family *Caliciviridae* in these buildings included HSalV, Norwalk Virus, Sal-FHB, and Sapporo virus. *Picornaviridae* species detected were Aichivirus A, Enterovirus C, and Parechovirus A (HPeV-A). AdV-40 and -41 (group F) and Human Bocavirus (HBoV), belonging to the *Parvoviridae* family, and Human polyomavirus 1 (HPyV-1 or BKPyV) and Human polyomavirus 2 (HPyV-2 or JCPyV) were also observed in school samples.

### University settings

Viral signatures identified in wastewater collected at the university settings in both cities showed remarkably similar patterns of viral excretion in their sewer outlets, with 60–70% of viral reads matching members of the family *Caliciviridae,* which also encompassed the highest diversity of viral species (HSalV, Sal-A, Sal-FHB and SapV). Additionally, reads matching members of the *Polyomaviridae,* JCPyV and BKPyV (15–20%), and the *Astroviridae* (AstV-1), (< 10%), were also consistently detected in both sites. Moreover, in site A university setting, there were reads belonging to families *Parvoviridae* (HBoV) (6%) and *Picornaviridae* (EV-B) (0,13%). Viral reads assigned to Alphapapillomavirus 2 (α-PV-2), Betapapillomavirus 2 (β-PV-2) and Gammapapillomavirus 7 (γPV-7) were also detected.

### Nursing homes

The viromes of the wastewater collected at the nursing homes were less diverse than those obtained from the other wastewater types. In nursing homes from both cities, the relative abundances of the viral families detected ranged between 15 and 30%. Sequences assigned to families *Polyomaviridae* (JCPyV, BKPyV and MCPyV) and *Papillomaviridae* (PV-7, β-PV-1, β-PV-2, and β-PV-3) were found in both locations, with *Papillomaviridae* having the highest diversity of viral species. In site A, viral reads matching the *Picornaviridae* family were identified (EV-C), while site B also presented few reads matching the Cercopithecine betaherpesvirus 5, also known as human cytomegalovirus (CMV), were unique to this type of setting.

## Discussion

In this study, WBE has been conducted at building-scale to study the human virome excreted by different aged populations. This has been achieved by analysing different types of wastewater using torpedo passive samplers previously reported as useful tools for viral detection and characterization in wastewater, especially at sites where composite samples are not always easy to obtain [13, 19]. Moreover, the use of TES allowed the detection of a high number of reads matching viral families that specifically infect humans.

Schools, university settings and nursing homes from two different cities were sampled and analysed for virome determination. The results, in terms of relative viral abundance, demonstrated similar viral excretion patterns for the same types of buildings in two different cities. It should be noted that some differences among buildings from the same type but from different cities arose, as well as variations within the same building sampled at different times. These differences were less pronounced when analysing results from the WWTP at different times (data not shown). Despite being derived from a restricted sample size, these results align with a recent study conducted by McCall and co-workers [15], who described higher variations among viral groups observed when comparing different buildings of the same type, as opposed to the variations observed among WWTPs. This observation appears reasonable when we consider the smaller number of individuals contributing to the wastewater virome at the building level. It also considers the possibility of small outbreaks being limited to very small communities, which are typically observed at the WWTP level, where the excretions of a significant percentage of the population are consolidated.

The wastewater virome obtained from the largest urban WWTP in Catalonia, analysed as the most representative sample of the complete human excreted virome in the studied geographical area, revealed a similar viral profile than reported in previous studies using the same TES approach [2, 19]. Astrovirus were the most prevalent viral family, followed by Human Bocavirus, Adenovirus F and Human Polyomaviruses. Interestingly, *Papillomaviridae* was the family with the highest number of different viral species. These findings align with the results reported by McCall and co-workers [15] who also reported *Astroviridae* and *Parvoviridae* as the predominant viral families detected in WWTP after applying a TES approach. Of the 37 distinct viral species detected in the WWTP, 18 were exclusively found in these WWTP samples and not in the building-scale wastewater. This probably indicates these viruses are excreted at low concentrations or by a small number of people and are detected when analysing samples integrating the shedding of a high number of contributors. On the other side, there were species belonging to the *Astroviridae* family which were solely identified in school wastewater, while some members of the *Papillomaviridae* family were exclusively detected in university settings and nursing homes but not in the WWTP. Furthermore, the relative abundances of certain viral species that were present at both building and WWTP-level were different, emphasizing the potential of this type of analysis to provide insights into the viral burden within different demographic groups. Given this finding, the significance of the abundance and observed occurrence trends of the detected viral species in the various samples analysed in this study will be further explored in the subsequent sections.

### Wastewater virome associated with persistent infections

Different families of viruses known to persistently infect humans have been found in the different wastewater samples analysed. A discernible trend reveals a higher excretion of these viral families among the elderly, followed by adults, and finally, by children. It is essential to emphasize that persistent infection does not necessarily culminate in clinical manifestations, although viruses that persistently infect humans can sporadically lead to pathological symptomatology.

#### Human adenoviruses

With more than 110 types described, different organ tropisms and a wide variety of clinical manifestations (revised in Rusiñol and Girones [1]), Human AdV are persistently excreted and make a significant contribution to the viral community present in sewage [2] being proposed as a human viral faecal indicator. The urban wastewater analysed contained reads that matched AdV-F, -A and -B. Among those AdV types identified (Additional file 1: Table S1), AdV-7, -21 and –B3 (Human mastadenovirus B), first isolated from young children, are associated with several respiratory diseases [20], while types A and F have been linked to gastrointestinal infections. Wastewater from the schools and the university settings wastewater only presented AdV-F (Adv-70 and -41)[21].

While both, elderly individuals and young children, are at a heightened risk of AdV infection [22], AdVs reads were not detected in the wastewater from nursing homes. The detection of human AdVs in wastewater have been correlated with the size of the contributing population being sometimes not detected in small size sewer systems [19, 23].

#### Human polyomaviruses

Human polyomaviruses, with 14 different members, are known to be transmitted among humans during the first years of live through close contact and are excreted by a significant percentage of the population in their urine, typically without recognizable clinical symptoms. Clinical manifestations of these viruses are primarily linked to deep immunosuppression. Among these, Merkel Cell polyomavirus (MCPyV) was discovered to cause Merkel Cell Skin Carcinoma linked to immunosuppression in elderly people and that ageing is a risk factor to develop it.

In this study, sequences matching BK, JC and MCPyV were detected in the WWTP influent. BK and JCPyV were detected in all building samples, whereas MCPyV reads were only observed in the nursing home. An observed seroprevalence increasing with age for JCPyV and decreasing for BKPyV has been previously described [24] and results here obtained are almost in fully agreement with this. The nursing homes studied are contributed to by a small number of people, which would explain the low number of JCPyV reads found. Its presence, along with the absence of human AdV, corroborates previously reported observations describing JCPyV as a faecal viral indicator [25] useful in small sewers [19, 23].

#### Human papillomaviruses

Within the *Papillomaviridae* family, β-PV and α-PV infect non-genital mucosa and skin. Their excretion has been linked to epithelial shedding in urine, and the presence of these viruses and their high diversity in the environment could be considered emerging [26, 27]. Members of the genus α-PV primarily infect oral and genital mucosa, as well as external genitals, and are associated with mucosal tumour development in humans [28]. Members of this genus are differentiated by their potential to induce cancer, classified as either low-risk Human Papillomavirus (LR-HPV) or high-risk Human Papillomavirus (HR-HPV), and are known to be causing persistent infections [29]. Their occurrence has been widely documented within the environment [30] reinforcing that investigating these viruses in these matrices is highly valuable for describing their prevalence in specific population groups.

Mucosal HPV infections have traditionally been considered sexually transmitted diseases, although could infect children through horizontal transmission, primarily from mother to child [31]. In this study, HPV reads were observed at WWTPs, university settings and nursing homes but not in school’s wastewater. In fact, this family was the more diverse found in urban wastewater with sequences matching 12 different viral species being HPV-90, a rare emergent LR-HPV, one of the more prevalent types found [32]. Out of the 12 different HPV species detected in urban sewage, 9 were not found at building-level wastewaters. β-PV 1 and 2 species (HPV-22, HPV-100 and HPV-75) and α-PV 7 (HPV-70) were described exclusively in nursing homes wastewater. HPV-70 has been linked to the development and progression of cervical intraepithelial neoplasia and other squamous intraepithelial lesions [33, 34] and to scrotal calcinosis development [34] and showed high prevalence among some unvaccinated male groups [35]. Lastly, α-PV s 7 (HPV-149) and α-PV 2 (HPV-28) have been detected exclusively in university settings, they are known to cause skin warts due to the cutaneous tropism of this species, unlike most α-PV which exhibits mucosal tropism [36, 37].

#### Herpesviruses

Regarding members of *Herpesviridae* family, which establish latency and persist for the life of the individual [38], human cytomegalovirus (CMV) is the only member of the family present in one of the nursing homes tested. CMV is the most common opportunistic pathogen in immunocompromised patients [39] but it can also infect immunocompetent individuals causing self-limiting illness resembling mononucleosis [40, 41]. Interestingly, this virus was also detected in a building wastewater by McCall [15].

### Wastewater virome associated with acute infections

Additionally, sequences ascribed to viruses belonging to families commonly associated with acute infections have been identified in the different wastewaters analysed. The overall observed trend suggests a higher excretion of these viruses by children and lower excretion by adults and the elderly. It is important to highlight that the presence of these viruses in wastewater indicates viral replication but does not necessarily translate to clinical manifestations, as these viruses can often cause subclinical infections, particularly in children.

#### Astroviruses

Human Astrovirus (HAstV) is one of the most important causes of viral gastroenteritis worldwide. Although described in faecal samples from adults, they are responsible for the 10% of acute viral gastroenteritis in children [42]. In this study, the only species common to all wastewater types analysed was Mamastrovirus-1, which decreased in reads number in agreement with the age of the contributor individuals (Fig. 3). HAstV sequences were consistently more prevalently detected in January samples (data not shown), aligning with their known seasonal pattern [43]. A deeper analysis of the sequences of Mamastrovirus-1, allowed a further classification into serotypes in some of the pooled samples (Additional file 1). Serotype HAstV-1 was identified in all samples which aligns with reported data indicating its high prevalence within the population [43]. These results agree with a prior study conducted in Spain, specifically targeting children under the age of 5 who suffered from acute gastroenteritis, and that study reported a 11.5% prevalence rate [44]. The abundance of reads corresponding to *Astroviridae* (AstV), and, in particular to AstV-1*,* were higher in school samples compared to other studied locations. AstV reads account for more than 80% of reads resulting from school wastewater analysis. This explains why the total numbers of reads from schools was higher than those obtained from the WWTP possibly indicating a higher viral load in the school environment due to elevated AstV excretion. AstV-6, AstV-8 and HMO Astrovirus A were only detected at WWTP-level. HAstV-2 and HAstV-3 reads have also been detected in school and university settings whereas HAstV-4 reads were only found in nursing home wastewater.

#### Caliciviruses

Norwalk virus is the only species belonging to the Norovirus genus (NoV). NoV infections are the leading cause of gastroenteritis outbreaks [45], are usually self-limiting in healthy patients although in immunocompromised individuals, elderly people and young children can cause severe complications [46, 47]. Norwalk species are divided into genogroups, that are divided into 30 genotypes. In this study, NoV was detected in schools, university settings and at the urban WWTP. A deeper analysis of the sequences obtained allowed a classification into genogroups in some samples (Additional file 1). Despite, genogroups GI, GII and GIV are prevalent human pathogens, only GI and II were found in this study. In particular, the GII.17 genotype, that was identified in the school from city B and at the WWTP, has gained prominence in recent years, overtaking GII.4 as the dominant strain in Asia [48] and has also been described in Europe [49–51].

Sapovirus is an important cause of diarrhoea in children, especially in countries where vaccines for rotavirus are available [52, 53]. They also have been identified as pathogen in adults, especially in immunocompromised individuals or those who live in nursing homes [54–56]. The results obtained showed Sapovirus reads in the wastewater collected from schools and university settings, which is consistent with previous reports indicating that it represents one of the highest burdens of disease in young children [57] as well as its association with sporadic and limited outbreaks [58].

All in all, results obtained suggest children and young adults are the main shedders of members of the *Caliciviridae* family while these viruses were not detected in nursing home wastewater suggesting no active infections or low excretion levels of these viruses at the time these analyses were conducted.

#### Coronaviruses

To date, seven human coronaviruses (HCoVs) have been described. In this study, only Human coronaviruses HKU1 (HCOV-HKU1) and SARS-CoV-2 were reported in the urban wastewater collected at the studied WWTP. HCOV-HKU1 has been associated with severe complications in vulnerable populations presenting cold-like symptoms [59] and SARS-CoV-2 with the Covid-19 [60]. These results suggest that if HCoV were excreted at building-level, which is likely, excretion levels were low and under the detection limits of techniques employed. It should be said that SARS-CoV2 was detected in all the buildings by applying qPCR [13] which is a more sensitive technique than NGS [18]. Recently, a specific probe-based capture panel designed for the specific sequencing of CoV has been successfully applied to the detection of a high diversity of human and animal CoV in wastewater (Martinez-Puchol et al., submitted to publication).

#### Parvoviruses

Human bocavirus (HBoV) is associated with respiratory symptoms and gastroenteritis in children [61] although its role as a causative agent has been not described yet [62]. HBoV is usually attributed to infectious in infants although it can affect children older than 5 years and adults with higher incidences in winter and spring [63, 64]. In this study, HBoV was present in school, university, and WWTP samples with the highest number of reads obtained from school samples in agreement with excretion patterns described for this virus and with previous studies regarding its presence in sewage [65–67]

#### Picornaviruses

Picornavirus sequences were detected in all the wastewater types analysed although WWTP samples presented the highest diversity of viral species suggesting excretion related to small outbreaks within small communities.

Aichivirus (AiV) is associated with childhood infections [68] responsible of a low proportion of gastroenteritis outbreaks [69], it has been detected in the stool of patients with gastroenteritis among different age groups in Spain [70] and in this study has been found in WWTP samples collected from schools and nursing homes.

The genus Cosavirus include five species of Human Cosavirus (HCoSV-A, B, D, E, F). It was first detected in stool samples of healthy children and children with non-polio acute flaccid paralysis [71, 72] and, later, in children with or without diarrhoea [73–76]. HCoSVs have also been found in sewage and rivers [67, 77, 78]. Despite their presence and diversity in sewage has been described [79], only few clinical studies have been published, showing a very low detection rate if any [80–83] that could explain why sequences matching HCoSV-A were only detected at the WWTP level but not at building-level.

The same happened with Hepatitis A virus (HAV) which usually cause self-limited infections being transmitted by faecal-oral route but also be spread by person-to-person transmission causing large epidemics [84]. The prevalence of HAV correlates with the socioeconomic status of the studied area [85]. In developed countries, where vaccination programs against HAV exist, the prevalence of HAV has decreased [86]. In this study, HAV was only detected at the WWTP-level. In Spain, specifically in Catalonia, vaccination against HAV is systemic and included in the vaccination schedule for children at 15 months old. Although it is not technically mandatory, it is widely practiced. We hypothesize that there was no detection at schools and university settings as children and young people are heavily immunised but people travelling to endemic areas, men having sex with men, prisoners, homeless and other susceptible collectives may contribute to the HAV observed at WWTP-level [87].

Both enterovirus (EV) and Human Parechovirus (HPeV) are picornaviruses causing a common infection in children. HPeV causes mainly respiratory and gastrointestinal symptoms [88] although in children have been associated with central nervous system infections [89]. EV have been associated with wide range of illness: from mild febrile illness, respiratory infection, gastroenteritis, meningitis, encephalitis, feet-hand-mouth disease and other pathologies [90]. However, EV is usually seen in older children and adults while HPeV is not frequent at these ages [91]. This aligns with our results since HPeV reads were only present at school and WWTP while EV reads were observed in all samples studied with differences in the distribution of the three species (A, B and C).

Human rhinoviruses (HRV) are the most prevalent human respiratory viruses and responsible for more than a half of cold-like illnesses each year. In this study, Rhinovirus B and C were only found in the WWTP and was no detected in building’s wastewater probably because the sampling was conducted in winter when influenza and respiratory syncytial viruses are predominant over HRV [92–94].

Salivirus (SaV) was first described from stool samples from children with gastroenteritis [95]. Its relationship with gastroenteritis remains still unclear [96] However, SaV has been detected worldwide in clinical samples and urban sewage [97, 98]. In our study, two species of SaV were identified: SaV-A at university settings and at the WWTP and SaV-FHB in schools, universities, and at the WWTP. These results agree with previous publications describing its association with infection in patients always under 20-year-old and its presence in children’s stool [99, 100]. SaV reads were the more abundant reads at the university settings even when comparing to WWTP, suggesting young adults as the SaV target population.

In summary, it can be hypothesized that the virome in human wastewater comprises a mosaic of viruses originating from a variety of sources. Some viruses may originate from individuals of various age groups, while others might be specific to certain populations. Additionally, our findings indicate that viruses associated with acute infections causing limited outbreaks are more frequently excreted by children than by the older population, and these viruses are typically detectable in WWTPs.

These viruses can also be detected at the building level during an ongoing outbreak at the time of sampling, or when asymptomatic individuals contribute to increased viral load in wastewater. On the other hand, certain viral species that are restricted to specific individuals or demographic groups, such as certain HPV or astrovirus species, may be more readily detectable at the building level and could become diluted to undetectable levels in the influent of larger wastewater treatment systems. Furthermore, the observed virome profiles can be valuable for understanding the epidemiology of these viruses, including their excretion patterns and transmission pathways.

## Conclusions

- Passive sampling, in the form of torpedo devices, was applied as a straightforward and efficient method for the wastewater virome characterization at a building-scale. TES provided a comprehensive list of viruses present at WWTP and building-level scale.
- The virome profiles observed in the two cities studied for the same type of building linked to a specific demographic group were similar suggesting different viral excretion profiles for each sewer system analysed.
- WWTP virome, analysed as a reference, presented astrovirus, particularly MAstV-1, as the more abundant viruses followed by HBoV, adenoviruses, polyomaviruses, picornaviruses and caliciviruses.
- Astrovirus (particularly AstV-1) predominated in school samples, while salivirus and human polyomaviruses JC and BK dominated in university settings. Nursing homes showed a more balanced distribution of viral families presenting papillomavirus and picornaviruses. Notably, viruses associated with immunosuppression, such as MCPyV and CMV.
- A discernible trend emerged: viruses associated with acute infections were more frequently detected in schools but less so in elderly residences, while viruses linked to persistent infections exhibit an inverse trend, being more commonly detected in elderly residences and less frequently in schools.
- Overall, the study provides insights into the diversity and distribution of human viruses in different types of wastewaters sources. The results highlight the potential of building-scale WBE as a tool for tracking communal infectious diseases. Such information can be useful in gaining insights into these infections and in designing targeted public health intervention strategies for specific populations.

## Methods

### Sample collection

While numerous WBE studies predominantly utilize composite samples gathered by automatic samplers at WWTP inlets, the advent of the COVID-19 pandemic has spurred the adoption of flexible passive sampling techniques [14, 16]. These approaches facilitate the monitoring and viral profiling of various waterborne pathogens within compact sewer catchments [16, 19].

Our study focussed on three distinct building types—schools, university settings, and nursery homes—in two cities in Catalonia, Northeast Spain, as outlined in Table 1. School buildings mainly accommodate students aged 3 to 12 but also a diverse range of users, including teachers, administrative staff, parents, guardians, maintenance, and support personnel, as well as participants in various after-school programs. Buildings at the university campus primarily cater to individuals aged 18 to 30, but given the dynamic nature of campuses, these spaces also host researchers, administrators, and other faculty members throughout the day.

**Table 1:** Sampling sites and building users description

Age group	City	Sampling sites	Population represented
3–12	A	School sewer outlet	500
18–30	A	University campus sewer outlet	100
> 65	A	Nursing home sewer outlet	300
3–12	B	School sewer outlet	500
18–30	B	University campus sewer outlet	400–1000
> 65	B	Nursing home sewer outlet	500
All ages	C	Inlet urban wastewater treatment plant	1.5 M

The studied nursing homes, accommodating 300 predominantly elderly residents as full-time inhabitants, represent facilities with comparatively lower user capacity. These residents constitute their primary users.

Additionally, wastewater from a third city (C), with an urban population of about 1.5 million, was obtained from the main WWTP inlet. This wastewater served as a reference for a comprehensive analysis of the excreted virome.

Buildings were sampled two/three times during January and March 2022 except for the nursery home at city A where only samples in March were collected (detailed in Additional file 1). The sampling was conducted using 3D printed torpedo-shaped passive sampling units (kindly donated by Prof. McCarthy) fitted with 2 electronegative membranes (EZ-Pak filters 0.45 µm, Merck Millipore). Torpedoes were deployed for 24 h at the outlet of each building sewer and at the inlet of the WWTP. They were retrieved and transported inside a plastic bag to the laboratory in a portable icebox.

### Elution of the viral particles, nucleic acid extraction and DNase treatment

Prior to nucleic acids (NA) extraction, the elution of the viruses adsorbed to the electronegative membranes was performed. Electronegative membranes were carefully introduced, using sterile tweezers, inside Power Bead Tubes (glass 0.1 mm Qiagen), 700 µl of glycine (0.25 N, pH = 8) were added and then bead-beating was applied for 30 s at 4 m/s using FastPrep-24™ (MP Bio, USA). Sample tubes were centrifuged for 1 min at 20.000×*g* and the resulting 60 µl supernatant was treated with Turbo DNase (Invitrogen, Carlsbad, CA, USA) for 1 h at 37 ºC. The QIAamp Viral RNA mini kit and the Qiacube Automatic system (Qiagen) were used for the NA extraction into a final volume of 50 µl.

### Sequence-independent, single-primer amplification (SISPA) and target enrichment sequencing (TES)

Nucleic acid extractions analysed in this study were pooled according to Additional file 2 and amplified before the sequencing library was constructed as previously described [101]. For this reason, viral sequences identified in each sampling site are the result of the viral sequences present in 2–3 different samples collected in January and March and are presented together to provide a more robust composition of a particular virome.

Briefly, NA were retrotranscribed and tagged using SuperScript IV enzyme (Invitrogen), random nonamer primers and Sequenase 2.0 (Applied Biosystems). The cDNA was then amplified to obtain enough cDNA for the next steps by following 25 PCR cycles using AmpliTaq Gold DNA polymerase (Applied Biosystems). The resulting product of the PCR was purified using Zymo DNA Clean & Concentration kit (Zymo research) and quantified using Qubit 2.0 and the Qubit dsDNA HS Assay Kit (Invitrogen).

Libraries were prepared following manufacture’s instruction using the KAPA HyperPlus Library Preparation Kit (KAPA Biosystems, Roche). Starting with 100 ng of amplified cDNA, enzymatic fragmentation, A-tailing and adapter’s ligation were conducted. Each sample was ligated with KAPA UDI Primer mixes (KAPA Biosystems Roche) and a clean-up was performed with the KAPA HyperPure Beads (KAPA Biosystems, Roche). The libraries were amplified with a LM-PCR of 7 cycles, purified and then quantified using Qubit 2.00 and the Qubit dsDNA HS Assay Kit (Invitrogen).

### Capture of viral sequences by VirCapSeq-VERT™ capture panel (Roche)

Libraries were pooled to obtain a final quantity of 1 µg. Then it was hybridised for 20 h with probes of the VirCapSeqVERT™ capture panel (Roche) that contains sequences of viruses infecting vertebrates using the HyperCap Target Enrichment Kit (Roche). The captured DNA was recovered using the HyperCap Bead Kit (Roche), amplified with 14 cycles of LM-PCR, purified (HyperPure Beads, Roche) and quantified with Qubit 2.0. Sequencing of the captured libraries was performed on an Illumina NextSeq2000 platform achieving up to 1000 M 150 × 2 pb pair-end reads.

The generated Pair-end FASTQ files were analysed using ID-seq, an open source and cloud-base bioinformatic tool [102]. Briefly, Illumina adapters, duplicates, low quality and complexity reads were cleaned using Trimmomatic [103] and CD-HITDUP tool v4.6.8 (CD-HIT, PRID:SCR 007105) [104] Reads were paired using the Paired-Reads Interactive Contig Extension (PRICE) computational package (PRICE, RRID:SCR 013063) [105] and the Lempel–Ziv–Wech (LZW) compression score. GSNAPL [106] and RAPsearch2 [107] were used for an assembly-based alignment to the NCBI nucleotide (nt) and non-redundant protein (nr) databases [108]. A minimum of 70% identity and > 100 nt length were considered for analysis. However, for human viruses, all species were assigned with alignments to the NCBI above 90% of identity.

Viral reads of *Adenoviridae* and *Papillomaviridae* longer than 100 bp were queried for similarity using BLASTN against the NCBI GenBank nucleotide collection database [108]. For specific characterization, Calicivirus and Enterovirus the Typing Tool developed by RIVM were used (version 2.0 and 0.1, respectively) [109].

### Statistical and diversity index calculations

The Shannon diversity indexes were calculated using Excel, as measures of the relative abundances of the species present in a sample. The formula to calculate the Sannon índex (H) is:

$$H = - \sum\nolimits_{i = 1}^{S} {p_{i} \cdot In\;} p_{i} .$$

where *S* is the total number of species in the community and* p*^*i*^ the proportion of reads belonging to the species.

### Supplementary Information

**Additional file 1**. List of Viral species and serotypes, serogroups or genotypes identified in each building type. Mean ID%: average percentage identity of alignments; Mean CV: the per cent of the reference accession that is covered by at least one contig; Coverage depth: indicates the average read depth across the length of the accession. Metagenomic metrics are mean values obtained at the different samples analysed per each building type and at the WWTP.

**Additional file 2**. Sample dates and pools of the nucleic acid extractions analysed. Each column corresponds to one pool.

## Data Availability

The datasets generated during the current study are available in zenodo under the DOI numbers: 10.5281/zenodo.8178269, 10.5281/zenodo.10077991, 10.5281/zenodo.10078619, 10.5281/zenodo.10149180, 10.5281/zenodo.10158645.

## Abbreviations

Adv: Adenovirus
AiV: Aichivirus
α-PV: Alphapapillomavirus
β-PV: Betapapillomavirus
BKPyV: BK polyomavirus
COVID-19: Coronavirus infectious disease
DNA: Deoxyribonucleic acid
EV: Enterovirus
EV-C: Enterovirus-C
γ-PV: Gammapapillomavirus
GI: Genogrup 1
HAV: Hepatitis A virus
HR-HPV: High-risk Human Papillomavirus
HAstV: Human Astrovirus
HMO-AstV-A: Human astrovirus A
HBoV: Human Bocavirus
HCoV: Human coronaviruses
HCoSV: Human Cosavirus
CMV: Human cytomegalovirus
HPV: Human Papillomavirus
HPyV: Human polyomavirus
HSalV: Human Salivirus
JCPyV: JC polyomavirus
LR-HPV: Low-risk Human Papillomavirus
AstV: Mamastrovirus
MCPyV: MC polyomavirus
NGS: Next-generation sequencing
NoV: Norovirus
NA: Nucleic acids
HPV: Human Papillomavius
HPeV-A: Paraechovirus A
qPCR: Quantitative Polymerase Chain Reaction
SaV: Salivirus
Sal-A: Salivirus A
Sal-FHB: Salivirus FHB
SapV: Sapovirus
SISPA: Sequence-independent, single-primer amplification
SARS-CoV-2: Severe acute respiratory syndrome coronavirus 2
TES: Target Enrichment approach
WWTP: Wastewater treatment plant
WBE: Wastewater-based epidemiology
