# Supplementary material for: Wastewater-based epidemiology applied at the building-level reveals distinct virome profiles based on the age of the contributing individuals
Source: Hum Genomics. 2024 Feb 1;18:10. doi: 10.1186/s40246-024-00580-1 (PMC10832175; doi:10.1186/s40246-024-00580-1)
Supplement: Supplementary file 1 — Additional file 1. List of Viral species and serotypes, serogroups or genotypes identified in each building type. Mean ID%: average percentage identity of alignments; Mean CV: the per cent of the reference accession that is covered by at least one contig; Coverage depth: indicates the average read depth across the length of the accession. Metagenomic metrics are mean values obtained at the different samples analysed per each building type and at the WWTP. [file 40246_2024_580_MOESM1_ESM.pdf]

Supplementary information 1: List of Viral species and serotypes, serogroups or genotypes identified in each building type. Mean ID%: avarage percentatge identity of alignments; Mean CV: the percent of the reference accession that is covered by at least one contig; Coverage depth: indicates the average read depth across the length of the accession. Metagenomic metrics are mean values obtained at the different samples analysed per each building type and at the WWTP.

Sampling site	Family	Viral specie	metagenomics metrics			Serotypes, serogroups or genotypes identified
			**Mean ID%**	**Mean CV%**	**Coverage depth**	
School	*Adenoviridae*	*Human mastadenovirus F*	99.9	7	2.2x	AdV-40, AdV-41
	Astroviridae	*Astrovirus MLB1*	97.5	5.9	0.33x	
		Mamastrovirus 1	99.35	97.5	2490x	HAstV-1, HAstV-3
		Mamastrovirus 3	98.9	-	-	
		Unclassified Mamastrovirus	93.1	-	-	
	Caliciviridae	*Human salivirus*	95.8	-	-	
		Salivirus FHB	98	-	-	
		Sapporo virus	97.55	25.6	22.5x	SapV-GI, -GII, -GIV
		Unclassified Sapovirus	97.8	-	-	
	Parvoviridae	*Human bocavirus*	99.8	52.6	96.1x	
	Picornaviridae	*Parechovirus A*	91.9	9.3	2.6x	
		Aichivirus A	97.3	-	-	
		Cardiovirus B	93	4.1	0.24x	
		Enterovirus C	93.2	4.8	0.44x	
		Norwalk virus	99.7	7.3	0.69x	NoVGII.17
	Polyomaviridae	*Human polyomavirus 1*	99.9	59.6	178.2x	
		Human polyomavirus 2	99.4	-	-	
University	*Adenoviridae*	*Human mastadenovirus F*	99.93	4.3	0.31x	
	Astroviridae	*Mamastrovirus 1*	98	5.4	0.48x	HAstV-1, HAstV-3
	Caliciviridae	*Human salivirus*	95.6	25	3.9x	
		Norwalk virus	99.7	23.6	3.5x	
		Salivirus A	94.1	-	-	
		Salivirus FHB	96.8	45	52x	
		Sapporo virus	96.4	5.6	0.72x	
	Papillomaviridae	*Alphapapillomavirus 2*	99.1	5.2	1.1x	HPV-28
		Betapapillomavirus 2	99.3	4.9	0.32x	HPV-9, HPV-110
		Gammapapillomavirus 7	98.6	28	2.2x	HPV-149
	Parvoviridae	*Human bocavirus*	99.3	10.9	4x	
	Picornaviridae	*Cardiovirus B*	92.35	-	-	
		Enterovirus B	94.6	-	-	
		Human cosavirus SH4	92.8	-	-	
	Polyomaviridae	*Human polyomavirus 1*	99.8	25	5.4x	
		Human polyomavirus 2	99.9	34.8	20x	
Nursing home	*Astroviridae*	*Mamastrovirus 1*	99.5	-	-	
	Herpesviridae	*Cercopithecine betaherpesvirus 5*	97.5	-	-	
	Papillomaviridae	*Alphapapillomavirus 7*	99.9	-	-	HPV-70
		Betapapillomavirus 1	98.8	3.1	0.39x	HPV-105
		Betapapillomavirus 2	99.3	-	-	HPV-22, HPV-100
		Betapapillomavirus 3	96.6	-	-	HPV-75
	Picornaviridae	*Cardiovirus B*	94.6	7.5	1x	
		Enterovirus C	94.2	39	6.5x	Coxsakie-A22, -A1
	Polyomaviridae	*Human polyomavirus 1*	98.8	-	-	
		Human polyomavirus 2	99.5	9.5	1.6x	
		Human polyomavirus 5	99.8	8.9	0.75x	
WWTP	*Adenoviridae*	*Human mastadenovirus A*	99.7	-	-	
		Human mastadenovirus B	99.7	-	-	AdV-7, -21 -B3
		Human mastadenovirus F	98.1	35.1	5.7x	
	Astroviridae	*Astrovirus MLB1*	96.8	15.7	2x	
		Mamastrovirus 1	98.6	88.1	182.8x	HAstV-1, HAstV-3, HAstV-4
		Mamastrovirus 6	98.2	-	-	
		Mamastrovirus 8	96.7	5.2	0.2x	
		HMO Astrovirus A	93.6	13.8	2.9x	
	Caliciviridae	*Human salivirus*	97.1	-	-	
		Norwalk virus	99.7	44.6	1.8x	NV-GI.P10, NV-GII.P7
		Salivirus A	96.05	-	-	
		Salivirus FHB	93.5	21	2.1x	
		Sapporo virus	99.3	63.3	5.1x	
		Unclassified Sapovirus	97.8	-	-	
	Coronaviridae	*SARS-CoV-2*	96.2	-	-	
		Human coronavirus HKU1	100	-	-	
	Papillomaviridae	*Alphapapillomavirus 14*	99.9	-	-	HPV-90
		Betapapillomavirus 1	97.1	14.2	2x	HPV-21, HPV-36
		Betapapillomavirus 2	100	8.8	0.8x	HVP-110
		Betapapillomavirus 3	97.9	-	-	HPV-76
		Betapapillomavirus 4	97.2	-	-	HPV-92
		Gammapapillomavirus 1	100	-	-	HPV-65
		Gammapapillomavirus 3	96.9	-	-	HPV-50
		Gammapapillomavirus 10	100	3.1	0.35x	HPV-133
		Gammapapillomavirus 11	95.3	-	-	HPV-169
		Gammapapillomavirus 19	94.3	4.8	0.29x	HPV-169
		Iotapapillomavirus 1	99.3	-	-	
		Pipapillomavirus 2	100	-	-	
	Parvoviridae	*Human bocavirus*	99.8	48.4	85.9x	
	Picornaviridae	*Aichivirus A*	98	16.3	3.8x	
		Aichivirus E	88.5	-	-	
		Cardiovirus B	95.65	-	-	
		Cardiovirus C	93.6	7.3	0.77x	
		Cosavirus A	93.5	6	0.49x	
		Enterovirus B	93.7	3.5	0.21x	
		Enterovirus C	91.5	6.7	1.2x	Coxsackie-A19
		Hepatovirus A	98.5	8.4	2.2x	
		Human cosavirus	94.1	-	-	
		Human enterovirus	94.2	-	-	
		Parechovirus A	97.2	-	-	
		Rhinovirus B	97.6	-	-	
		Rosavirus B	98.9	3	0.1x	
	Polyomaviridae	*Human polyomavirus 1*	97.4	55.6	28.4x	
		Human polyomavirus 2	99.8	26.5	6.1x	
		Human polyomavirus 5	99.8	9.7	2.6x	
