# Supplementary material for: Wastewater-based epidemiology applied at the building-level reveals distinct virome profiles based on the age of the contributing individuals
Source: Hum Genomics. 2024 Feb 1;18:10. doi: 10.1186/s40246-024-00580-1 (PMC10832175; doi:10.1186/s40246-024-00580-1)
Supplement: Supplementary file 2 — Additional file 2. Sample dates and pools of the nucleic acid extractions analysed. Each column corresponds to one pool. [file 40246_2024_580_MOESM2_ESM.pdf]

Supplementry information 2: Sample dates and pools of the nucleic acid extractions. Each column correspond to one pool

**School**				**University**				**Nursing home**			**WWTP**	
A		B		A		B		A		B	C	
Pool 1	Pool 2	Pool 3	Pool 4	Pool 5	Pool 6	Pool 7	Pool 8	Pool 9	Pool 10	Pool 11	Pool 12	Pool 13
10/1/2022	10/3/2022	13/1/2022	17/3/2022	10/1/2022	10/3/2022	10/1/2022	17/3/2022	13/1/2022	10/3/2022	22/3/2022	18/1/2022	10/3/2022
13/1/2022	17/3/2022	20/1/2022	22/3/2022	18/1/2022	17/3/2022	13/1/2022	22/3/2022	20/1/2022	17/3/2022	10/3/2022	20/1/2022	22/3/2022
20/1/2022	22/3/2022					20/1/2022			22/3/2022	17/3/2022		
